# Good prognosis for follicular lymphoma with estrogen receptor α‐positive follicular dendritic cells

**DOI:** 10.1002/hon.2730

**Published:** 2020-04-13

**Authors:** Rintaro Ohe, Hong‐Xue Meng, Akane Yamada, Naing Ye Aung, Takanobu Kabasawa, Yuka Tamura, Aya Utsunomiya, Nobuyuki Tamazawa, Ichiro Kawamura, Takumi Kitaoka, Kazushi Suzuki, Ryo Yanagiya, Tomomi Toubai, Kenichi Ishizawa, Mitsunori Yamakawa

**Affiliations:** ^1^ Department of Pathological Diagnostics Yamagata University Faculty of Medicine Yamagata Japan; ^2^ Department of Pathology Harbin Medical University Cancer Hospital Harbin China; ^3^ Department of Neurology, Hematology, Metabolism, Endocrinology and Diabetology Yamagata University Faculty of Medicine Yamagata Japan

**Keywords:** estrogen receptor alpha, follicular dendritic cell, follicular lymphoma, FLIPI, Prognosis

## Abstract

Follicular lymphoma (FL) has a meshwork of follicular dendritic cells (FDCs). We previously demonstrated the presence of estrogen receptor alpha (ERα)^+^CD23^+^ FDCs in grades 1‐2 FL. The significance of FDCs as a prognostic factor in FL remains unknown. The current study aimed to compare clinicopathological features, including prognosis, between FL with and without ERα^+^ FDCs. This study evaluated the clinicopathological significance of ERα expression in 70 FL patients by immunostaining. The presence of *ERα* mRNA on FDCs from 5 FL patients was confirmed by CD21/*ERα* double staining (immunohistochemistry and in situ hybridization). We defined patients with frequent ERα expression as the ERα^high^ group and those with infrequent ERα expression as the ERα^low^ group. Thirty‐two patients were assigned to the ERα^high^ group (45.7%), and 38 patients were assigned to the ERα^low^ group (54.3%). Both overall survival (OS) and progression‐free survival (PFS) were significantly better in the ERα^high^ group than in the ERα^low^ group (OS, log‐rank, *P* = .0465; PFS, log‐rank, *P* = .0336). Moreover, high ERα expression on FDCs was an independent prognostic factor for OS in both the univariate ([hazard ratio] HR, 0.163; *P* = .0260) and multivariate (HR, 0.050; *P* = .0188) analyses and for PFS in both the univariate (HR, 0.232; *P* = .0213) and multivariate (HR, 0.084; *P* = .0243) analyses. *ERα* mRNA expression was detected in CD21^+^ FDCs within the neoplastic follicles of FL patients. In conclusion, a neoplastic follicular microenvironment with ERα‐positive FDCs might affect the grade and presence of the follicular pattern of FL and improve patient prognosis.

## 1 INTRODUCTION

Follicular lymphoma (FL) is a germinal center (GC)‐derived lymphoma^1^, ^2^ that is frequently followed by an indolent clinical course.^3^, ^4^ As prognostic factors, the Follicular Lymphoma International Prognostic Index (FLIPI)^5^ and FLIPI2^6^ are commonly used. Recently, m7‐FLIPI,^7^ which includes the mutation status of 7 genes, and progression of disease within 2 years (POD24 or POD2),^8^ which is defined as relapse or progression of FL within 24 months (2 years) after diagnosis, have been used as prognostic factors. In addition, various proteins of neoplastic cells themselves, such as CD5,^9^ GNA13,^10^ and FOXP‐1,^11^ are considered prognostic factors. In the microenvironment, it is unclear whether tumor‐associated macrophages and programmed cell death‐1^+^ cell infiltration are associated with prognosis.^12^, ^13^ Concerning follicular dendritic cells (FDCs), a tight CD21^+^ FDC meshwork frequently promotes transformation into large B‐cell lymphoma,^3^ although the extent of Ki‐M4p^+^ or the CD23^+^ FDC meshwork is not associated with treatment outcome or survival time.^14^

We previously demonstrated that the number of ERα^+^ cells was positively correlated with the width of the CD23^+^ FDC meshwork in both nonneoplastic GC and neoplastic follicle, and that estrogen receptor alpha (ERα)^+^ CD23^+^ FDCs supported the neoplastic follicular microenvironment of grades 1‐2 (G1‐2) FL, but not G3 FL, suggesting the possibility of the usefulness of antiestrogen therapy against FL.^15^

This study first compared clinicopathological features and prognosis between FL patients with more and less frequent ERα^+^ FDCs and investigated the significance of ERα^+^ FDCs in the FL microenvironment.

## 2 MATERIALS AND METHODS

### 2.1 Patients and samples

We investigated 70 tissue samples from FL patients before treatment. Pathological diagnoses were determined at Yamagata University Hospital and Yonezawa City Hospital in Japan and Harbin Medical University Cancer Hospital in China between 2003 and 2018 using the rituximab‐containing regimen. Specific FL variants and subtypes, including testicular FL, in situ FL, duodenal‐type FL, pediatric‐type FL, and primary cutaneous follicle center lymphoma, were excluded from this study. The FL specimens were classified as G1‐2 (n = 35), G3A (n = 22), or G3B (n = 13) in accordance with the WHO Classification Revised Fourth Edition.^16^ The histological pattern of these cases was classified as follicular pattern (n = 52) and another pattern (n = 18).^16^ Tissues were fixed in 10% neutral‐buffered formalin for 6 to 12 hours at room temperature, embedded in paraffin (formalin‐fixed paraffin‐embedded; FFPE), and used for hematoxylin‐eosin staining, immunostaining (IHC), and in situ hybridization (ISH). This study was approved by the Research Ethics Committee of Yamagata University Faculty of Medicine (H29‐343 & 2019‐108) and the Research Ethics Committee of Harbin Medical University Cancer Hospital (KY2016‐25) and was performed in accordance with the Declaration of Helsinki.

### 2.2 Evaluation of ERα IHC

IHC was performed as previously described,^17^ and a specific antibody for ERα (EP1; rabbit IgG, DAKO, Agilent Technologies, Santa Clara, California) was used. IHC was performed using an Autostainer Link 48 system (Agilent Technologies). ERα reactivity was estimated as previously described.^15^ Briefly, cells positive for ERα were counted in five neoplastic follicles for each case. In FL specimens with diffuse proliferation, positive reactions were counted in five high‐power fields (HPFs) at the area of diffuse proliferation. [Modified]: We revised the mean value of the number of ERα‐positive cells/HPF (ERα/HPF, 40x magnification, 0.159 mm^2^), which referred to the histological grading of the WHO Classification Revised Fourth Edition^16^ as follows: ERα/HPF = mean value of the number of ERα^+^ cells/area of neoplastic follicles (mm^2^) × 0.159 mm^2^ (×40 magnification). Our previous study demonstrated that the number of ERα was substitutable as a width of CD23^+^ FDC meshwork in nonneoplastic and neoplastic follicles, and indicated that the majority of ERα^+^ FDCs simultaneously expressed CD23.^15^ Therefore, we estimated ERα/HPF as a semi‐quantitative marker of the width of ERα^+^ CD23^+^ FDC meshwork in this study.

### 2.3 IHC of other proteins

To confirm the immunophenotype of FL, IHC was performed using antibodies specific for CD10 (56C6; mouse IgG1, DAKO, Agilent Technologies, Santa Clara, California), CD20 (L26; IgG2aκ, DAKO, Agilent Technologies), BCL2 (124; mouse IgG1κ, DAKO, Agilent Technologies), BCL2 (E17; rabbit IgG, Abcam, Cambridge, UK), BCL6 (PG‐B6p; mouse IgG1κ, DAKO, Agilent Technologies), MUM1 (MUM1p; mouse IgG1κ, DAKO, Agilent Technologies), CD21 (1F8; IgG1κ, DAKO, Agilent Technologies), CD23 (DAK‐CD23; mouse IgG1κ, DAKO, Agilent Technologies), and CD23 (SP23; rabbit IgG, Nichirei, Tokyo, Japan) by using an Autostainer Link 48 system (DAKO, Agilent Technologies). The reactivity of CD10, CD20, BCL2, BCL6, and MUM1 was considered positive if more than 30% of the neoplastic cells were positive. The area of the neoplastic follicle was determined from images and estimated as the gross area by ImageJ as previously described.^15^, ^18^, ^19^ The CD23^+^ FDC pattern was divided into 2 groups: none/dim or focal/marginal^4^/diffuse.

### 2.4 Double staining (IHC and ISH) of serial sections

To identify ERα‐expressing cells, double staining (IHC/ISH) was performed using the FFPE serial sections of the 5 FL patients with only follicular pattern and the 5 FL patients with diffuse pattern, and one uterine endometrioid carcinoma patient (as a positive control) as previously described,^20^ with minor modifications. Briefly, in one section, CD21 immunostaining was performed using an Autostainer Link 48 system (DAKO, Agilent Technologies) and visualized with 3,3′‐diaminobenzidine. For the ISH of other sections, digoxigenin‐labelled riboprobes encoding human *ERα* (human estrogen receptor 1, 854 bp, nucleotides 3001‐3845) and an ISH kit (Genostaff, Tokyo, Japan) were used. Probes were hybridized for 24 hours at 60°C, incubated with anti‐digoxigenin‐alkaline phosphatase (Roche Diagnostics, Indianapolis, Indiana) for 1 hour at room temperature, and visualized with BCIP/NBT Substrate System (DAKO, Agilent Technologies) for 24 hours at room temperature. The visual images for CD21 IHC and *ERα* ISH were overlaid by using ImageJ.^18^, ^19^ Sense probes were used as negative controls. Nuclear staining was performed with Kernechtrot.

### 2.5 Statistical analysis

To compare the clinicopathological characteristics of FL, the *χ* ^2^ test, Fisher's exact test or the Mann‐Whitney test was used. The endpoint of overall survival (OS) was defined as the time from diagnosis until all‐cause death, and the endpoint of progression‐free survival (PFS) was defined as the time from diagnosis until relapse due to FL.^9^, ^10^ Kaplan‐Meier curves of OS and PFS were drawn and compared by the log‐rank test. To propose prognostic factors, univariate and multivariate Cox proportional hazards regression models were used. Statistical analyses were performed using JMP version 14 (SAS Institute, Tokyo, Japan). Differences with *P* values <.05 were considered significant in each analysis.

## 3 RESULTS

### 3.1 IHC and ISH analyses of ERα

**FIGURE 1:**
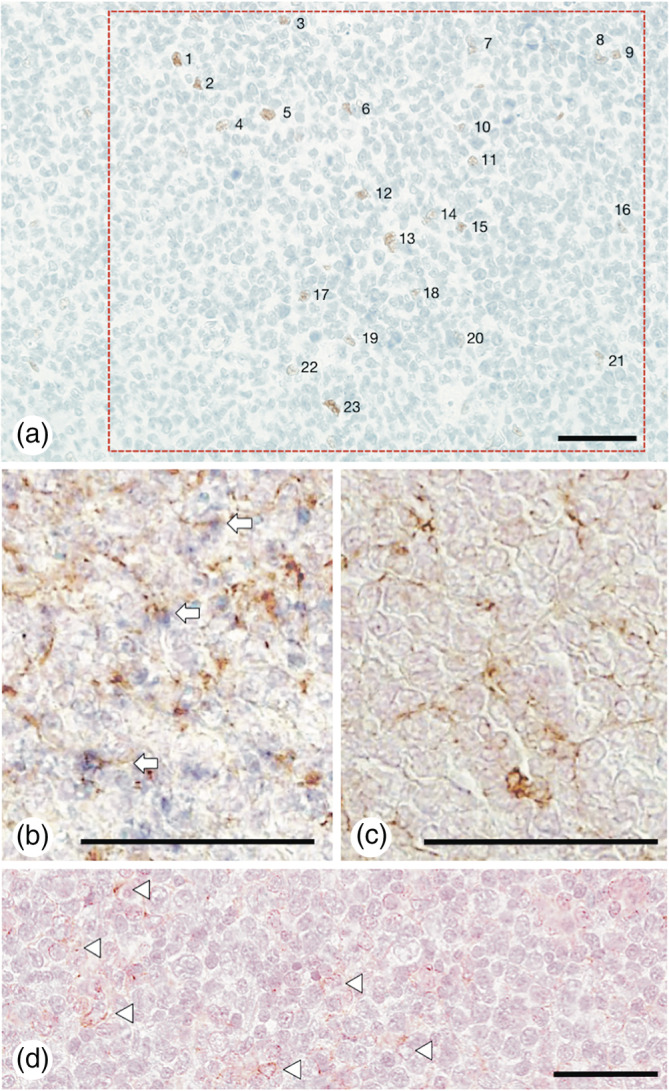
Immunohistochemistry (IHC) of the estrogen receptor alpha (ERα) protein and in situ hybridization (ISH) of *ERα* mRNA in follicular lymphoma (FL). IHC shows that ERα is expressed on follicular dendritic cells (FDCs) in a neoplastic follicle of G1‐2 FL (a). The area in the red dashed frame is 0.1 mm^2^. The number of ERα‐positive cells in the frame is 23. Therefore, the number of ERα‐positive cells/high‐power field (HPF; ×40 magnification, 0.159 mm^2^) was 36.57, and formalin‐fixed paraffin‐embedded tissue sections from FL patients were used for double staining of CD21 (IHC) and *ERα* (ISH). The left panel (b) shows hybridization with an antisense riboprobe, and the right panel (c) shows hybridization with a sense riboprobe (control) on serial sections. The expression of *ERα* mRNA was detected in FDCs within the neoplastic follicle of FL patients. CD21^+^ FDCs were stained with 3,3′‐diaminobenzidine (brown), and *ERα* mRNA^+^ cells were stained with NBT‐BCIP (dark blue); double stained CD21^+^/*ERα* mRNA^+^ FDCs are shown as arrows in (b). The expression of *ERα* mRNA was not detected in diffuse proliferation area of FL patients, although CD21^+^ FDCs was slightly detected (d; Arrowhead). Nuclear staining was performed with Kernechtrot. Bars, 50 μm

In the IHC analysis, ERα expression was detected mainly in FDCs in neoplastic follicles, as previously reported.^15^ OS, PFS, and ERα/HPF were compared between the ERα high‐expression (ERα^high^) and ERα low‐expression (ERα^low^) groups. As a result, most of the significant differences in OS and PFS were observed when FL patients were divided into 2 groups: ≥ 3 ERα/HPF and <3 ERα/HPF. Therefore, patients with ≥3 ERα/HPF were as assigned to the ERα^high^ group, and the other patients were assigned to the ERα^low^ group (<3 ERα/HPF). Thirty‐two patients (32/70, 45.7%) were assigned to the ERα^high^ group, and 38 patients (38/70, 54.3%) were assigned to the ERα^low^ group. ERα/HPF were 10.2 ± 6.45 in the ERα^high^ group (Figure 1a) and 0.20 ± 0.54 in the ERα^low^ group (*P* < .0001). In sequential IHC/ISH, the expression of *ERα* mRNA was detected in FDCs within the neoplastic follicles of 5 FL patients (Figure 1b,c). *ERα* mRNA was not detected in diffuse proliferation area of 5 FL patients (Figure 1d) and CD21^+^ FDC meshwork hardly/did not exist in this area.

### 3.2 Relationship between the frequency of ERα expression and the clinicopathologic features of FL

The comparison of each clinicopathological feature between the ERα^high^ and ERα^low^ groups is shown in Table 1. Regarding the histological grade, there was more G1‐2 FL in the ERα^high^ group than in the ERα^low^ group and more G3 FL in the ERα^low^ group than in the ERα^high^ group (*P* < .0001). Moreover, the ERα^high^ group had a higher frequency of the follicular proliferative pattern than the ERα^low^ group (*P* < .0001). Immunohistochemically, the ERα^high^ group had a higher frequency of the CD23^+^ FDC meshwork than the ERα^low^ group (*P* < .0001). In the initial therapy, there was a more watchful wait in the ERα^high^ group than in the ERα^low^ group (*P* = .0077). However, other features, such as age, sex, FLIPI, t(14;18)(q32;q21), the expression of other markers (CD10, BCL2, BCL6, and MUM1), the initial therapy regimen, and the rate of complete response to initial therapy were not significantly different between the ERα^high^ and ERα^low^ groups.

**TABLE 1:** ERα expression on follicular dendritic cell (FDC) in follicular lymphoma (70 cases)

	ERα/HPF ≥ 3 (n/N [%])		
	Positive cases	Negative cases	
	(N = 32)	(N = 38)	*P* value
Clinical features			
Age (median [range]) (years)	58 (40‐82)	60 (38‐80)	.2289^a^
Age >60 y	12/32 (37.5)	18/38 (47.4)	.4051
Male/female	13/19	19/19	.4328
Ann Arbor stage III‐IV	17/31 (54.8)	19/34 (55.9)	.9326
Bulky mass > 6 cm	6/31 (19.4)	4/34 (11.8)	.6150
Lymph node >4 regions	14/31 (45.2)	13/34 (38.2)	.5714
Bone marrow involvement	8/24 (33.3)	5/31 (16.1)	.1364
Hemoglobin level < 12 mg/dL	8/31 (25.8)	9/34 (26.5)	.9515
Evaluated LDH level	11/31 (35.5)	19/34 (55.9)	.0994
β2‐microglobulin	14/21 (66.7)	17/22 (77.3)	.4383
FLIPI, high risk	12/31 (38.7)	13/34 (38.2)	.9687
t(14;18)(q32;q21)	14/20 (70.0)	6/10 (60.0)	.8911
Histological features			
Grade 1–2 (baseline G3A & 3B)	30/32 (93.8)	5/38 (13.2)	<.0001^b^
Follicular pattern (baseline including diffuse pattern)	31/32 (96.9)	21/38 (55.3)	<.0001^b^
Immunohistochemistry			
CD10 expression	28/32 (87.5)	32/38 (84.2)	.7452
BCL2 expression	32/32 (100)	33/38 (86.8)	.0581^b^
BCL6 expression	29/32 (90.6)	30/38 (79.0)	.2083
MUM1 expression	4/32 (12.5)	12/38 (31.6)	.1078
CD23 FDC pattern (baseline dim/none)	27/32 (84.4)	9/37 (24.3)	<.0001
Initial therapy			
Watchful wait	9/32 (28.2)	3/35 (8.57)	.0077^b^
Chemotherapy			
R‐containing regimen	23/32 (71.9)	28/35 (80.0)	.3319
Others	0/32 (0)	1/35 (2.86)	1^b^
Radiation therapy	0/32 (0)	2/35 (5.71)	.4934^b^
Chemotherapy & radiation therapy	0/32 (0)	1/35 (2.86)	1^b^
Others	0/32 (0)	0/35 (0)	NA
Complete response to initial therapy	16/23 (69.6)	26/33 (78.8)	.6380
Median follow up (median [range]) (months)	32 (7‐85)	57 (10‐137)	

Abbreviations: FLIPI, follicular lymphoma international prognostic index; HPF, high‐power field (×40 magnification, 0.159 mm^2^); LDH, lactate dehydrogenase; NA, not available; R, rituximab.

a Mann‐Whitney test.

b Fisher exact test.

### 3.3 Comparison of survival between the ERα^high^ and ERα^low^ groups

**FIGURE 2:**
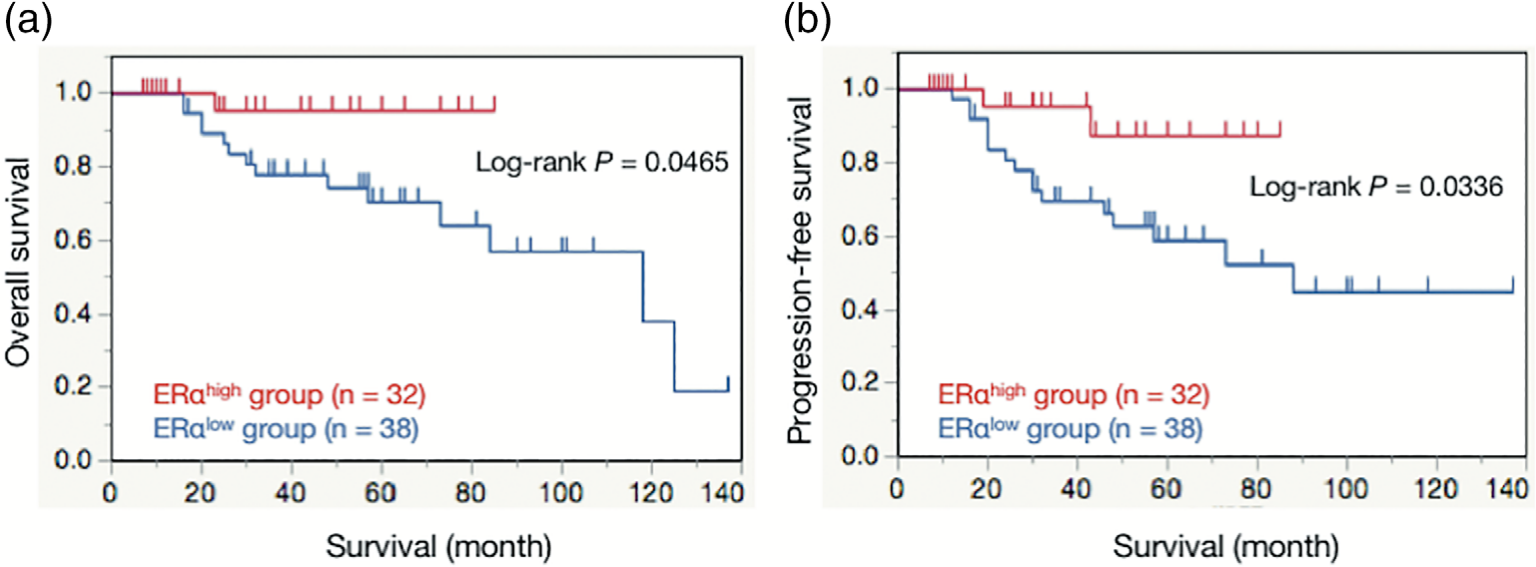
Prognostic analyses of overall survival (OS) and progression‐free survival (PFS) according to estrogen receptor alpha (ERα) expression in FL patients. The ERα^high^ group had significantly better OS than the ERα^low^ group (log‐rank, *P* = .0465) (a). The ERα^high^ group had significantly better PFS than the ERα^low^ group (log‐rank, *P* = .0336) (b). In the ERα^high^ group, the number of ERα‐positive cells was ≥3/HPF (×40 magnification, 0.159 mm^2^); in the ERα^low^ group, the number of ERα‐positive cells was <3/HPF

Kaplan‐Meier curves of OS and PFS are shown in Figure 2. The ERα^high^ group had a significantly better prognosis for both OS and PFS than the ERα^low^ group (OS, log‐rank, *P* = .0465; PFS, log‐rank, *P* = .0336).

### 3.4 Univariate and multivariate analyses of OS and PFS

The results of the univariate and multivariate analyses of OS are shown in Table 2. In the univariate analysis, age (≥61 y; hazard ratio (HR), 6.520 [95% confidence interval (CI) [1.814‐23.44]], *P* = .0011), Ann Arbor stage III‐IV (HR, 15.32 [95% CI, [1.971‐119.0]], *P* = .0003), high serum LDH (HR, 4.264 [95% CI [1.205‐20.17]], *P* = .0235), number of lymphadenopathy areas (> 4; HR, 4.601 [95% CI [1.443‐17.30]], *P* = .0098), hemoglobin (<12 g/dL; HR, 4.497 [95% CI [1.396‐14.49]], *P* = .0131), high‐risk FLIPI (HR 8.743 [95% CI [2.585‐39.65]], *P* = .0004), CD10 positivity (HR, 0.178 [95% CI [0.053‐0.623]], *P* = .0086), and ERα/HPF (≥3; HR, 0.163 [95% CI [0.009‐0.834]], *P* = .0260) were significantly associated with OS. Moreover, in the multivariate analysis, high‐risk FLIPI was an independent poor prognostic factor for OS (HR, 15.63 [95% CI [3.579‐102.4]], *P* < .0001), and ERα/HPF were an independent good prognostic factor for OS (HR, 0.050 [95% CI [0.002‐0.606]], *P* = .0188).

**TABLE 2:** Univariate and multivariate analysis for overall survival and progression‐free survival of follicular lymphoma cases

	Overall survival						Progression‐free survival					
	Univariate analysis			Multivariate analysis			Univariate analysis			Multivariate analysis		
Parameter	HR (95% CI)		*P* value	HR (95% CI)		*P* value	HR (95% CI)		*P* value	HR (95% CI)		*P* value
Age ≥ 61 (years)	6.520	(1.814‐23.44)	.0011				4.850	(1.805‐15.24)	.0015			
Sex: male (baseline female)	2.194	(0.744‐6.466)	.1418				2.072	(0.815‐5.636)	.1260			
Ann Arbor, stage III‐IV (baseline I‐II)	15.32	(1.971‐119.0)	.0003				10.33	(2.876‐65.90)	<.0001			
High serum LDH (baseline standard value)	4.264	(1.205‐20.17)	.0235				2.330	(0.836‐7.503)	.1075			
Number of lymphadenopathy areas >4	4.601	(1.443‐17.30)	.0098				5.981	(2.153‐19.14)	.0006			
Bulky mass > 6 cm	0.645	(0.035‐3.344)	.6565				1.279	(0.199‐4.667)	.7541			
Bone marrow involvement	1.617	(0.351‐5.554)	.4971				2.146	(0.590‐6.270)	.2231			
Hemoglobin <12 g/dL	4.497	(1.396‐14.49)	.0131				2.464	(0.832‐6.670)	.0991			
FLIPI, high‐risk (baseline low‐intermediate risk)	8.743	(2.585‐39.65)	.0004	15.63	(3.579‐102.4)	<.0001	9.157	(3.156‐33.00)	<.0001	11.00	(3.319‐46.61)	<.0001
CD10 positivity	0.178	(0.053‐0.623)	.0086				0.334	(0.125‐1.094)	.0685			
BCL2 positivity	0.547	(0.146‐3.535)	.4643				0.844	(0.239‐5.347)	.8253			
BCL6 positivity	2.425	(0.621‐16.27)	.2224				0.760	(0.283‐2.400)	.615			
MUM1 positivity	3.191	(0.981‐10.47)	.0536				1.581	(0.541‐4.198)	.3828			
Histological grade 3A & 3B (baseline grade 1‐2)	1.744	(0.582‐6.365)	.3311	3.351	(0.410‐24.10)	.2386	2.456	(0.878‐8.677)	.0893	3.754	(0.526‐20.98)	.1698
Follicular pattern (baseline another pattern including diffuse pattern)	0.434	(0.143‐1.355)	.1451	1.877	(0.461‐7.568)	.3684	0.328	(0.127‐0.848)	.0222	0.925	(0.270‐2.933)	.8965
CD23 FDC marginal and diffuse pattern (baseline none and dim pattern)	1.007	(0.342‐2.969)	.9890				0.759	(0.275‐1.984)	.5751			
ERα/HPF ≥ 3	0.163	(0.009‐0.834)	.0260	0.050	(0.002‐0.606)	.0188	0.232	(0.037‐0.825)	.0213	0.084	(0.009‐0.708)	.0243

Abbreviations: CI, confidence interval; ERα, estrogen receptor alpha; FDC, follicular dendritic cell; FLIPI, follicular lymphoma international prognostic index; HPF, high‐power field (×40 magnification, 0.159 mm^2^); HR, hazard ratio; LDH, lactate dehydrogenase.

The results of the univariate and multivariate analyses of PFS are also shown in Table 2. In the univariate analysis, age (HR, 4.850 [95% CI [1.805‐15.24]], *P* = .0015), Ann Arbor stage III‐IV (HR, 10.33 [95% CI (2.876‐65.90]], *P* < .0001), number of lymphadenopathy areas (HR, 5.981 [95% CI [2.153‐19.14]], *P* = .0006), high‐risk FLIPI (HR, 9.157 [95% CI [3.156‐33.00]], *P* < .0001), follicular pattern (another pattern, including the diffuse pattern, at baseline) (HR, 0.328 [95% CI [0.127‐0.848)], *P* = .0222), and ERα/HPF (HR, 0.232 [95% CI [0.037‐0.825]], *P* = .0213) were significantly associated with PFS. Moreover, in the multivariate analysis, high‐risk FLIPI was an independent poor prognostic factor for PFS (HR, 11.00 [95% CI [3.319‐46.61]], *P* < .0001), and ERα/HPF were an independent good prognostic factor for PFS (HR, 0.084 [95% CI [0.009‐0.708]], *P* = 0.0243).

## 4 DISCUSSION

There was a higher frequency of G1‐2 FL, the follicular pattern and CD23^+^ FDCs in the ERα^high^ group than in the ERα^low^ group in this study (Table 1). The FDC immunophenotype of G1 FL resembles that of the light zone (LZ).^21^ We previously described that ERα^+^ CD23^+^ FDCs were distributed both in the LZ of GC and in the neoplastic follicle from G1‐2 FL, unlike G3 FL,^15^ indicating that the microenvironment of the ERα^high^ group is similar to the LZ of GC supported by ERα^+^CD23^+^ FDCs.

To the best of our knowledge, this study was the first to reveal an association between high ERα expression and a good prognosis in FL. Established prognostic factors, including FLIPI^5^ and FLIPI2,^6^ are used to judge the pre‐treatment status. Two additional prognostic factors were recently reported: m7‐FLIPI^7^ is used to determine the pre‐treatment status, and minimal residual disease^22^ and POD24^8^ are used to determine the post‐treatment status. However, their roles as prognostic factors in different histological grades, except for G3B, have become questionable during current therapies.^16^ Immunohistochemical expression of CD5,^9^ GNA‐13,^10^ and FOXP‐1 ^11^ was recently reported as another prognostic factor of FL. The roles of intrafollicular tumor‐associated macrophages and programmed cell death‐1 expression as prognostic factors have not yet been established.^12^, ^13^ In particular, the extent of the FDC meshwork is not associated with OS^14^ and could be either a good or poor prognostic factor of transformation.^3^, ^23^ These results suggest that the relation between FDCs and prognosis remains ambiguous. However, we first described frequent ERα expression on FDCs in FL as an independent good prognostic factor in FL patients. Our results suggest that high ERα expression might be a candidate prognostic factor for FL. Moreover, ERα/HPF can be counted in the same field that pathologists usually examine to judge the histological grade of FL. Furthermore, ERα expression can be easily estimated with IHC because it is used worldwide for breast cancer hormone therapy.

The role of ERα expression on FDCs in FL and how hormone therapy can be applied for FL should be investigated in the future. We previously suggested that hormone therapy tended to decrease ERα expression and the CD21^+^ CD23^+^ FDC meshwork in non‐neoplastic axillary lymph nodes.^15^ Tamoxifen, an ERα antagonist, regulates both non‐neoplastic and malignant hematopoietic cells^24^ and promotes the apoptosis‐inducting effect of ceramide for leukemia and other cancers.^25^ Furthermore, tamoxifen is also considered a G protein‐coupled estrogen receptor (GPER) agonist and suppresses the proliferation of Jurkat cells, a T‐ALL cell line expressing GPER.^26^ GPER promotes the survival of mantle cell lymphoma cells.^27^ Therefore, our results suggest that GC‐derived lymphomas, including FL,^1^, ^2^ might be driven by apoptosis via the application of tamoxifen because FDCs prevent FL cells against apoptosis.^4^, ^28^ We should investigate GPER expression on lymphomas accompanied by the FDC meshwork in a further study.

We acknowledge several limitations of this study. First, it is necessary that future studies carefully evaluate the significance of ERα^+^ FDCs. For instance, pER (Ser118), as a predictive marker of a good response to tamoxifen,^29^ can be analyzed on FDCs in FL to more effectively adapt anti‐hormone therapy. Second, it is important to investigate ERα^+^ FDCs in other GC‐derived lymphomas, such as angioimmunoblastic T‐cell lymphoma, classic Hodgkin lymphoma, and nodular lymphocyte predominant Hodgkin lymphoma.^1^, ^2^

In conclusion, this study is the first to demonstrate the different clinicopathological characteristics between ERα^high^ and ERα^low^ patients with FL. These results suggest that a neoplastic follicular microenvironment with ERα‐positive FDCs might affect the histological grade and presence of the follicular pattern of FL and indicate a good prognosis for FL patients.

## CONFLICT OF INTEREST

All authors declare no conflict of interest.
